# Rhythmic profile of memory T and B-cells along childhood and adolescence

**DOI:** 10.1038/s41598-023-48115-3

**Published:** 2023-11-28

**Authors:** Joaquim Pedro Brito-de-Sousa, Maria Luiza Lima-Silva, Ismael Artur da Costa-Rocha, Luiz Roberto Alves de Oliveira Júnior, Ana Carolina Campi-Azevedo, Vanessa Peruhype-Magalhães, Josiane da Silva Quetz, Jordana Grazziela Alves Coelho-dos-Reis, Christiane Costa-Pereira, Cristiana Couto Garcia, Lis Ribeiro do Vale Antonelli, Cristina Toscano Fonseca, Jandira Aparecida Campos Lemos, Juliana Vaz de Melo Mambrini, Elaine Maria Souza-Fagundes, Andréa Teixeira-Carvalho, Ana Maria de Caetano Faria, Angelica Oliveira Gomes, Karen Cecília de Lima Torres, Olindo Assis Martins-Filho

**Affiliations:** 1 Programa de Pós-graduação em Imunologia e Parasitologia Aplicadas, Universidade Federal de Uberlândia, Uberlândia, MG Brazil; 2 Instituto René Rachou, Fundação Oswaldo Cruz (FIOCRUZ-Minas), Avenida Augusto de Lima, 1715, Barro Preto, Belo Horizonte, MG 30190-002 Brazil; 3 Departamento de Bioquímica e Imunologia, Instituto de Ciências Biológicas, Universidade Federal de Minas Gerais, Belo Horizonte, MG Brazil; 4 Universidade Professor Edson Antônio Velano, UNIFENAS, Belo Horizonte, MG Brazil; 5 Laboratório de Virologia Básica e Aplicada, Departamento de Microbiologia, Instituto de Ciências Biológicas, Universidade Federal de Minas Gerais, Belo Horizonte, MG Brazil; 6 Secretaria Municipal de Saúde de Belo Horizonte, Belo Horizonte, MG Brazil; 7 Universidade Federal do Triângulo Mineiro, Uberaba, MG Brazil

**Keywords:** Lymphocytes, B cells, T cells, Immunological memory

## Abstract

Immunobiography describes the life-long effects of exogenous or endogenous stimuli on remodeling of immune cell biology, including the development of memory T and B-cells. The present study aimed at investigating the rhythms of changes in phenotypic features of memory T and B-cells along childhood and adolescence. A descriptive-observational investigation was conducted including 812 healthy volunteers, clustered into six consecutive age groups (9^Mths^–1^Yr^; 2^Yrs^; 3–4^Yrs^; 5–7^Yrs^; 8–10^Yrs^; 11–18^Yrs^). Immunophenotypic analysis of memory T-cell (CD4^+^ and CD8^+^) and B-cell subsets were performed by flow cytometry. The results pointed out that memory-related biomarkers of T and B-cells displayed a bimodal profile along healthy childhood and adolescence, regardless of sex. The first stage of changes occurs around 2^Yrs^, with predominance of naive cells, while the second and more prominent wave occurs around the age 8–10^Yrs^, with the prevalence of memory phenotypes. The neighborhood connectivity profile analysis demonstrated that the number of correlations reaches a peak at 11–18^Yrs^ and lower values along the childhood. Males presented higher and conserved number of correlations when compared to females. Altogether, our results provide new insights into immunobiography and a better understanding of interactions among the cellular subsets studied here during childhood and adolescence.

## Introduction

As the individuals grow and age, they undergo a remodeling in several body systems. These changes impact organ functions, tissue structures, as well as cellular interactions^1^. Regarding the immune system, alterations can be noted in the memory T and B-cell compartments, and they are influenced by previous infections, vaccinations, microbiome, food-derived and inhaled antigens, in a biological phenomenon known as immunobiography^2,3^.

The susceptibility to infections observed in newborns is caused by the immaturity of their lymphocytes, in addition to the low number of effector and memory T-cells, poor signaling of B-cell receptors and low production of antibodies and cytokines. There are three adaptive mechanisms to circumvent this susceptibility and increase newborn survival: polymorphonuclear elevations, antibody-mediated transplacental immunity, and antibody transfer during breastfeeding. Two of the three mechanisms are composed of elements of the maternal adaptive immune system that compensate for the less developed immune functions at birth^4^.

Thus, the immune system matures during childhood^2^, and as a result, changes in lymphoid organs lead to a replacement of naive T and B-cells in the periphery by activated experienced cells. Therefore, development and aging promote cumulative exposure to foreign antigens with accumulation of memory cells^5^. The plasticity and dynamicity of the developing immune system during childhood assists in the generation of several new connections with different pathogens, influencing and reverberating in the immune response later in life^6^.

By assessing the expression of quantitative trait loci considering single-nucleotide polymorphisms that associate with transcript abundance of a nearby gene (cis-eQTLs) in several human immune cell types and considering T-cell activation profiles, 12,254 unique genes have been identified. These genes contribute to the high complexity to the immune system, and their expression can be impacted by biological factors such as age, sex, and sex hormones^7–9^.

There is a variety of different cells throughout the organism, particularly in the immune system, that interact with each other, either directly or through soluble mediators. Our group has previously demonstrated the existence of distinct waves, rhythms, and dynamic network connectivity of serum soluble mediators along healthy aging with differences in magnitude and timing depending on sex difference. Regardless of sex, the childhood (3–10^Yrs^), or the first wave, is the period that exhibited the highest neighborhood connectivity amongst the soluble mediators, as well as a broad increase in these mediators. However, males displayed the second wave earlier (31–40^Yrs^ to 51–60^Yrs^), when compared with females (41–50^Yrs^ to 61–70^Yrs^)^10^.

This study aimed at investigating the changes in the cellularity of the immune system to better understand the changes in T and B-cell subsets and their rhythmic variations during childhood and adolescence. Our hypothesis is that the rhythm of memory responses during childhood induces differential patterns that have the potential to cause impact on the development of immune system throughout the individual's lifetime and, therefore, influencing its immunobiography.

## Results

### Rhythmic profile of memory T and B-cells along childhood and adolescence

**Figure 1:**
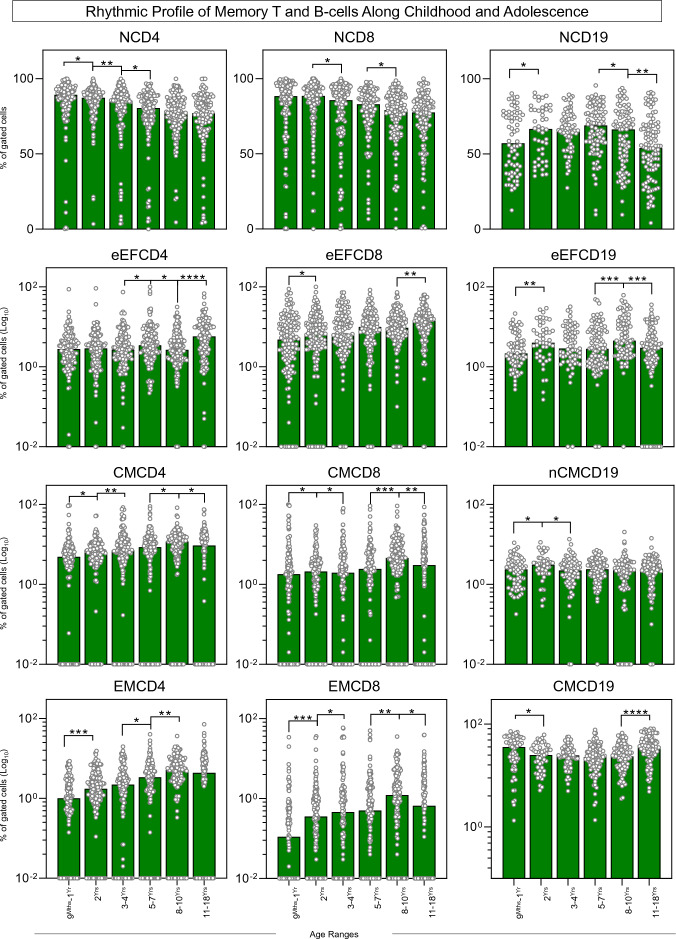
Rhythmic profile of memory T and B-cells along childhood and adolescence. The overall profile of memory T-cell subsets (CD4^+^ and CD8^+^) and B-cells was characterized in peripheral blood samples from healthy children and adolescents (ALL (Green color bar), n = 812), categorized into distinct age groups (9^Mths^–1^Yr^, n = 135; 2^Yrs^, n = 147; 3–4^Yrs^, n = 129; 5–7^Yrs^, n = 133; 8–10^Yrs^, n = 140 and 11–18^Yrs^, n = 128). Immunophenotypic analysis of NAIVE (N), Early EFFECTOR (eEF), CENTRAL (CM) and EFFECTOR MEMORY (EM) T-cell subsets as well as NAIVE (N), Early EFFECTOR (eEF), NON-CLASSICAL MEMORY (nCM) and CLASSICAL MEMORY (CM) B-cells was carried out by flow cytometry as described in Material and Methods. The results are shown as scattering distribution of individual data of relative frequency of gated cells (% within CD4^+^, CD8^+^ or CD19^+^ gated cells) over bars of median values for each age range. Comparative analysis between pairs of adjacent age groups was carried out by Mann–Whitney test and significant differences at p < 0.05 underscored by connecting lines for comparisons with the immediately earlier age range and the statistical significance identified by *, **, ***, and **** for p values ≤ 0.05; ≤ 0.01, ≤ 0.001, and ≤ 0.0001, respectively.

The phenotypic profile of memory T and B-cell subsets were assessed in healthy subjects at distinct age groups along childhood and adolescence (Fig. 1).

Comparative analysis between pairs of adjacent age groups in the general population (ALL) demonstrates an evident cycle of increase in several biomarkers at the age of 2^Yrs^, including: NCD19, eEFCD8, eEFCD19, CMCD4, CMCD8, nCMCD19, EMCD4, EMCD8 along with decreased frequencies of NCD4 and CMCD19, as compared to the baseline (age 9^Mths^–1^Yr^). By the age of 3–4^Yrs^, increased CMCD4 and EMCD8, and decreased NCD4, NCD8, CMCD8 and nCMCD19 were observed, as compared to the age 2^Yrs^. Around age 5–7^Yrs^, increased eEFCD4 and EMCD4 and a decrease of NCD4 were identified in comparison to the adjacent age range 3–4^Yrs^. Thereafter, around age 8–10^Yrs^, a second rise in cellular memory phenotypes was observed with increased percentages of eEFCD19, CMCD4, CMCD8, EMCD4, EMCD8, accompanied by decreases in NCD8, NCD19 and eEFCD4 in comparison to age 5–7^Yrs^ (Fig. 1).

Conversely, at adolescence (11–18^Yrs^), waning of a cluster of biomarkers composed of NCD19, eEFCD19, CMCD4, CMCD8 and EMCD8 was identified as compared to childhood (8–10^Yrs^), regardless of the increases in eEFCD4, eEFCD8 and CMCD19 (Fig. 1).

Overall, the rhythmic variations in memory-related biomarkers of T-cell subsets (CD4^+^ and CD8^+^) and B-cells revealed a bimodal profile of changes with a first cycle around 2^Yrs^ and a second and more prominent wave occurring by the age of 8-10^Yrs^. An inverted profile between NCD19 and CMCD19 was observed along development with an expansion of naive cells around 3–4^Yrs^ and 5–8^Yrs^, contrasting with classical memory, which presented higher values at 9^Mths^–1^Yr^ and 11–18^Yrs^ (Fig. 1).

### Rhythmic profile of memory T and B-cells along childhood and adolescence according to sex

**Figure 2:**
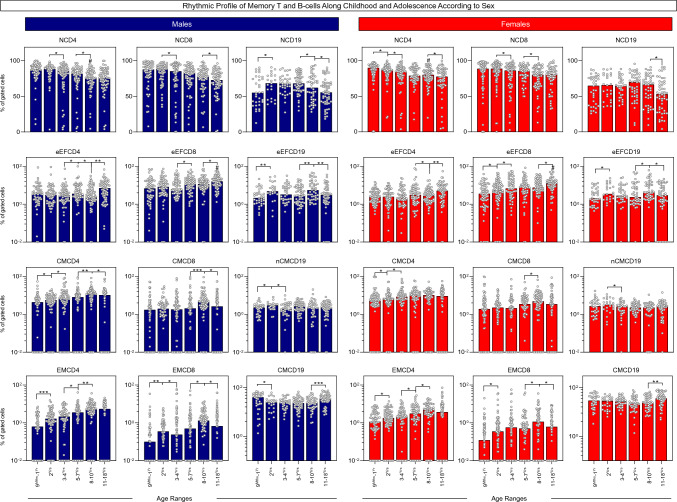
Rhythmic profile of memory T and B-cells along childhood and adolescence according to sex. The overall profile of memory T-cell subsets (CD4^+^ and CD8^+^) and B-cells was characterized in peripheral blood samples from healthy children and adolescents, categorized according to sex (Males (Blue color bar), n = 408 and Females (Red color bar), n = 404) followed by age stratification into distinct groups (9^Mths^–1^Yr^, n = 62, n = 73; 2^Yrs^, n = 67, n = 80; 3–4^Yrs^, n = 67, n = 62; 5–7^Yrs^, n = 72, n = 61; 8–10^Yrs^, n = 71, n = 69 and 11–18^Yrs^, n = 69, n = 59, respectively). Immunophenotypic analysis of NAIVE (N), Early EFFECTOR (eEF), CENTRAL (CM) and EFFECTOR MEMORY (EM) T-cell subsets as well as NAIVE (N), Early EFFECTOR (eEF), NON-CLASSICAL MEMORY (nCM) and CLASSICAL MEMORY (CM) B-cells was carried out by flow cytometry as described in Material and Methods. The results are shown as scattering distribution of individual data of relative frequency of gated cells (% within CD4^+^, CD8^+^ or CD19^+^ gated cells) over bars of median values for each age range. Comparative analysis between males and females at matching age ranges was carried out by Mann–Whitney test and significant differences at p < 0.05 underscored by #. Comparative analysis between pairs of adjacent age groups was carried out by Mann–Whitney test and significant differences at p < 0.05 underscored by connecting lines for comparisons with the immediately earlier age range and the statistical significance identified by *, **, ***, and **** for p values ≤ 0.05; ≤ 0.01, ≤ 0.001, and ≤ 0.0001, respectively.

The dynamic of changes in memory-related phenotypes was further assessed in males and females at distinct ages along childhood and adolescence (Fig. 2). Comparative analysis between males and females at matching age ranges revealed significant differences only for NCD4 at 8–10^Yrs^ and for eEFCD8 at 11–18^Yrs^ (Fig. 2, # symbols). These findings demonstrated that the analysis of rhythmic profile along adjacent ages represent a more valuable approach to identify sex-related changes in memory T and B-cells along childhood development than the comparative analysis at matching age ranges.

Comparative analysis between pairs of adjacent age groups demonstrated that at the age of 2^Yrs^, males presented increased percentages of NCD19, eEFCD19, CMCD4, nCMCD19, EMCD4 and EMCD8, and a decrease of CMCD19, while females exhibited increased frequencies of eEFCD8, eEFCD19, CMCD4, EMCD4, and EMCD8 with a decrease of NCD4, as compared to intragroup early age range 9^Mths^–1^Yr^. At the age of 3–4^Yrs^, both sexes displayed a quite similar profile with increased percentages of CMCD4 in males and eEFCD8 and CMCD4 in females, along with a decrease of NCD4, NCD8, nCMCD19 and CMCD19 in males, and NCD4, NCD8 and nCMCD19 in females. By the age of 5–7^Yrs^, it was observed increased percentages of eEFCD8 and EMCD4 in males and EMCD4 in females. A second wave of changes in memory-related biomarkers was identified for both sexes at age 8–10^Yrs^, as demonstrated by increased frequencies of eEFCD19, CMCD4, CMCD8, EMCD4 and EMCD8 in males, and eEFCD19, CMCD8, EMCD4 and EMCD8 in females, along with a decrease of NCD4, NCD19 and eEFCD4 in males, and NCD8 and eEFCD4 in females (Fig. 2).

Conversely, at adolescence (11–18^Yrs^), a decrease in several memory-related biomarkers was also identified in males (NCD8, NCD19, eEFCD19, CMCD4, CMCD8, and EMCD8) and in females (NCD4, NCD19, eEFCD19, and EMCD8), along with the increases in eEFCD4, eEFCD8 and CMCD19 observed in both sexes (Fig. 2).

Similar to the data observed for the general population (ALL), the rhythmic changes observed for males and females followed a bimodal profile with a first peak around age 2^Yrs^ and a second and more prominent crest observed at age 8–10^Yrs^ (Fig. 2).

### Predicted probability of changes in memory T and B-cells along healthy childhood and adolescence

**Figure 3:**
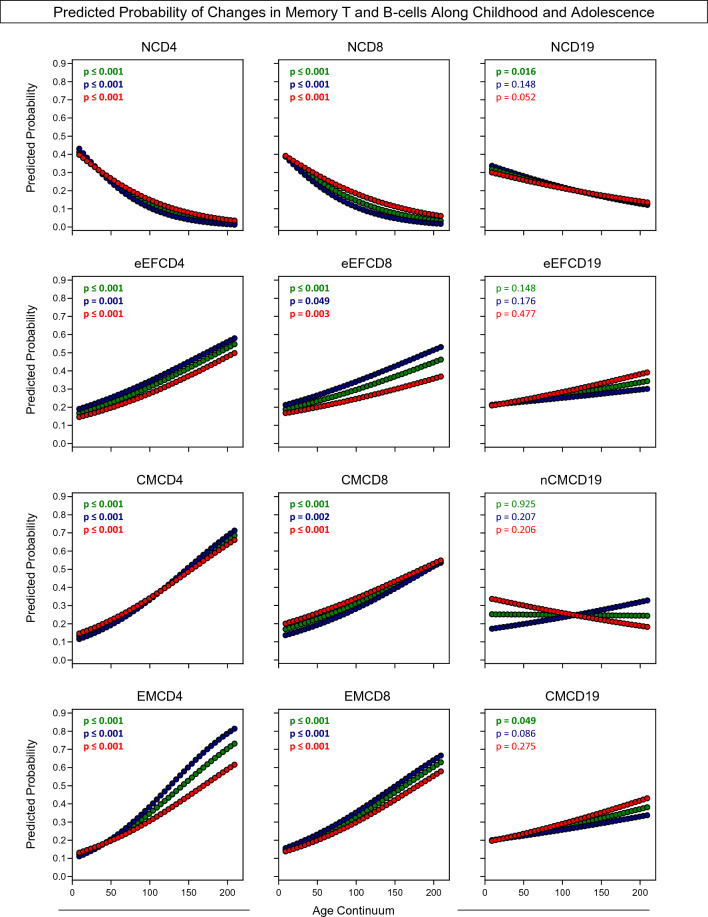
Predicted probability of changes in memory T and B-cells along childhood and adolescence. The association between memory T-cell subsets (CD4^+^ and CD8^+^) and B-cells with childhood and adolescence was carried for healthy children and adolescents (ALL (Green circle), n = 812), further categorized according to sex (Males (Blue circle), n = 408 and Females (Red circle), n = 404). Data categorization was performed based on cut-offs established at the 75th percentile of each data set empirical distribution. The strength of association between the frequency of memory T-cell subsets and B-cells and age as well as putative variations by sex was assessed by logistic regression model as described in Material and Methods. Predicted probabilities were estimated by age and the final data shown as scattering distribution of changes in memory T-cell subsets and B-cells along age continuum (months) and significant differences considered at p < 0.05. The p values for the general population (ALL) and data stratification by sex (Males and Females) are provided in the figure. Significant p values are underscored in bold.

Predicted probabilities were calculated by logistic regression to assess the strength of association between memory T and B-cell subsets with ageing during childhood and adolescence (Fig. 3). The predicted probabilities of subjects to present a given phenotype of memory T or B-cell subset in high frequency were estimated according to age continuum (months) with further data stratification by sex. Data analysis showed that naive T and B-cells (NCD4, NCD8, NCD19) had a continuous decrease in values along healthy childhood and adolescence. Conversely, memory T-cell subsets (eEFCD4; eEFCD8, CMCD4, CMCD8, EMCD4, and EMCD8) showed a continuous increase in values of predict probability along healthy childhood and adolescence. While a progressive decrease in NCD19 and an increase of CMCD19 was observed for ALL group, no differences were observed when the study population was segregated by sex. No difference in eEFCD19 and nCMCD19 were observed (Fig. 3).

### Panoramic signatures of memory T and B-cells along childhood and adolescence

**Figure 4:**
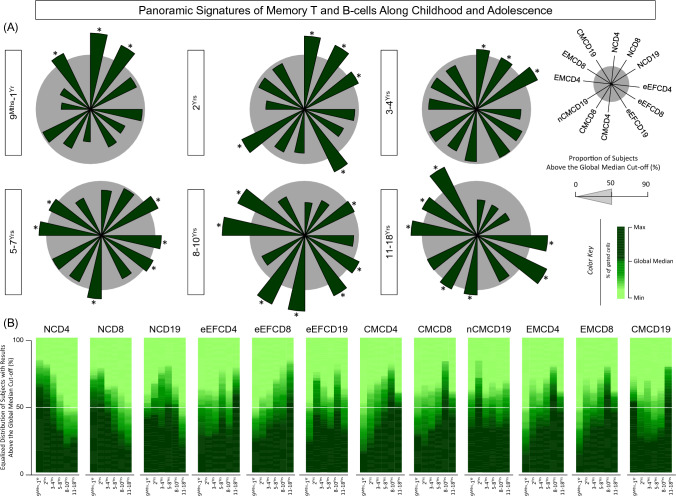
Panoramic signatures of memory T and B-cells along childhood and adolescence. (**A**) The overall signature of memory T-cell subsets (CD4^+^ and CD8^+^) and B-cells was assembled for healthy children and adolescents (ALL (Green color bar), n = 812), categorized according to distinct age groups (9^Mths^–1^Yr^, n = 135; 2^Yrs^, n = 147; 3–4^Yrs^, n = 129; 5–7^Yrs^, n = 133; 8–10^Yrs^, n = 140 and 11–18^Yrs^, n = 128). Immunophenotypic analysis of NAIVE (N), Early EFFECTOR (eEF), CENTRAL (CM) and EFFECTOR MEMORY (EM) T-cell subsets as well as NAIVE (N), Early EFFECTOR (eEF), NON-CLASSICAL MEMORY (nCM) and CLASSICAL MEMORY (CM) B-cells was carried out by flow cytometry as described in Material and Methods. Signatures were assembled by first converting the original data, expressed as continuous variables (% of gated cells), into categorical results reported as proportion (%) of subjects with results above the cut-off edges (global median values of each cell subset). The final data are shown in radar chart, with each axis representing one memory cell subset. Cell phenotypes with proportion higher than the 50th percentile (gray zone) were underscored by *. (**B**) Heatmaps constructs were built to further illustrate the rhythmic distribution patterns of memory T-cell subsets and B-cells. Data are expressed as equalized distribution of subjects with results above the global median cut-off based on the color key provided in the figure. A white line set on the 50^th^ percentile of data distribution was used to define the overall rhythm of signatures of memory T-cell subsets and B-cells.

Aiming at providing a panoramic snapshot of memory T-cell (CD4^+^ and CD8^+^) and B-cell subsets along healthy childhood and adolescence, the original results expressed as continuous variables were converted into categorical data to estimate the proportion of subjects with values above the global median cut-off defined for each memory cell subset (Fig. 4).

NCD4, NCD8 and CMCD19 phenotypes were observed in high proportion of subjects at age 9^Mths^–1^Yr^. While NCD4 and NCD8 still remained elevated at age 2^Yrs^, an increased proportion of subjects also presented elevated levels of NCD19, eEFCD19, and nCMCD19 by the age of 2^Yrs^. Around age 3–4^Yrs^, a selective increase in the naive cell subsets (NCD4, NCD8, and NCD19) was still observed. Inversely, at age 5–7^Yrs^, eEFCD4, eEFCD8, CMCD4, EMCD4, and EMCD8 cells increased, which highlight them as relevant memory-related biomarkers, along with NCD19. Thereafter at age 8–10^Yrs^, the set of memory-related phenotypes observed expanded with the inclusion of eEFCD19 and CMCD8 at age 5–7^Yrs^.

Noteworthy was that a massive increase in most memory-related phenotypes emerged at adolescence (11–18^Yrs^) with increased proportion of subjects with elevated levels of eEFCD4, eEFCD8, CMCD4, CMCD8, EMCD4, EMCD8, and CMCD19, except for eEFCD19 and nCMCD19 (Fig. 4A).

Aiming at providing a complementary landscape profile of changes in the overall signature of memory T and B-cells, heatmaps were assembled to underscore the cell phenotypes with increased proportion at each age range (Fig. 4B). The heatmap profiles pointed out the existence of a clear dichotomic profile among memory T-cell subsets. At early age there was a predominance of naive T-cell subsets (NCD4 and NCD8) and a shift towards memory-related phenotypes (eEFCD4, eEFCD8, EMCD4, EMCD8, CMCD4 and CMCD8), with marked expansion at late childhood (8–10^Yrs^) with slight remodeling at adolescence (11–18^Yrs^). Conversely, the signature of memory B-cell phenotypes exhibited a distinct rhythm. While NCD19 displayed a unimodal peak at 3-4^Yrs^ and 5-7^Yrs^, CMCD19 exhibited an opposite profile with higher values at 9^Mths^–1^Yr^ and 11–18^Yrs^. Moreover, the unimodal peak of nCMCD19 at age 2^Yrs^ contrasted with the bimodal profile of eEFCD19 with waves at 2^Yrs^ and 8–10^Yrs^ (Fig. 4B).

**Figure 5:**
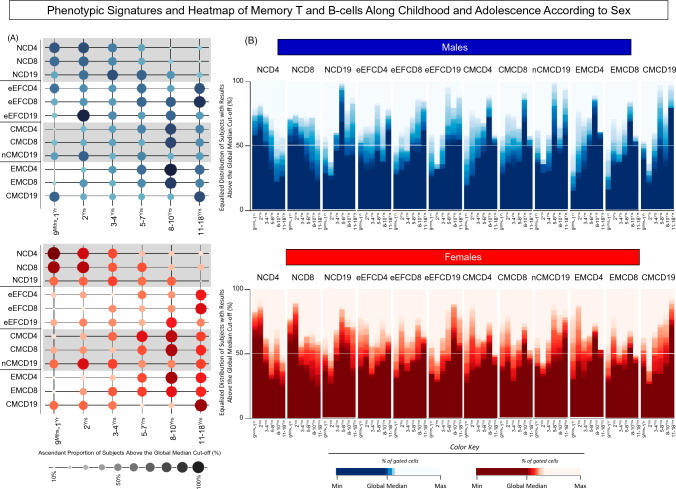
Signatures of memory T and B-cells along childhood and adolescence according to sex. (**A**) The overall signature of memory T-cell subsets (CD4^+^ and CD8^+^) and B-cells was assembled for healthy children and adolescents, categorized according to sex (Males (Blue color bar), n = 408 and Females (Red color bar), n = 404) followed by age stratification into distinct groups (9^Mths^–1^Yr^, n = 62, n = 73; 2^Yrs^, n = 67, n = 80; 3–4^Yrs^, n = 67, n = 62; 5–7^Yrs^, n = 72, n = 61; 8–10^Yrs^, n = 71, n = 69 and 11–18^Yrs^, n = 69, n = 59, respectively). Immunophenotypic analysis of NAIVE (N), Early EFFECTOR (eEF), CENTRAL (CM) and EFFECTOR MEMORY (EM) T-cell subsets as well as NAIVE (N), Early EFFECTOR (eEF), NON-CLASSICAL MEMORY (nCM) and CLASSICAL MEMORY (CM) B-cells was carried out by flow cytometry as described in Material and Methods. Signatures were assembled by first converting the original data, expressed as continuous variables (% of gated cells), into categorical results reported as proportion (%) of subjects with results above the cut-off edges (global median values of each cell subset). The final data are shown in orbital chart, with each circle representing one memory cell subset with size and color proportional to the frequency (%) of subjects with results above the cut-off edges. (**B**) Heatmaps constructs were built to further illustrate the rhythmic distribution patterns of memory T-cell subsets and B-cells. Data are expressed as equalized distribution of subjects with results above the global median cut-off based on the color key provided in the figure. A white line set on the 50th percentile of data distribution was used to define the overall rhythm of memory T-cell subsets and B-cells signatures.

The analysis of signature patterns of memory T and B-cells according to sex was also performed (Fig. 5). Data analysis demonstrated that similarly to what was observed for the general population (ALL), higher proportion of males and females presented elevated levels of naive cell subsets at early ages with an increase of memory cell subsets at late age groups (Fig. 5A). Heatmap analysis further corroborated these findings, demonstrating that naive cell subsets were predominant at early childhood and memory phenotypes at late age groups (Fig. 5B).

### Integrative networks of memory T and B-cells along healthy childhood and adolescence

**Figure 6:**
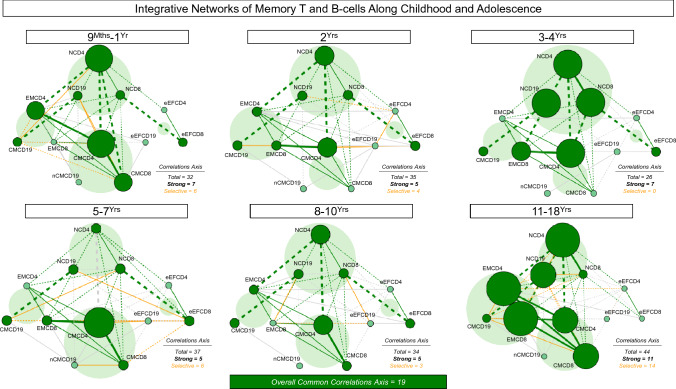
Integrative networks of memory T and B-cells along childhood and adolescence. Comprehensive networks were built for memory T-cell subsets (CD4^+^ and CD8^+^) and B-cells from healthy children and adolescents (ALL (Green color bar), n = 812), categorized into distinct age groups (9^Mths^–1^Yr^, n = 135; 2^Yrs^, n = 147; 3–4^Yrs^, n = 129; 5–7^Yrs^, n = 133; 8–10^Yrs^, n = 140 and 11–18^Yrs^, n = 128). Data analyses were carried out by Spearman rank tests as described in Material and Methods. Networks were built using cluster layouts comprising four groups of memory cell phenotypes, including: NAIVE (N), Early EFFECTOR (eEF), CENTRAL/NON-CLASSICAL (CM/nCM) and EFFECTOR/CLASSICAL MEMORY (EM/CM) subsets. Connecting edges identify positive (continuous line) and negative (dashed line) correlations. The line thickness illustrates the correlation strength, comprising weak/moderate (“r” scores from |0.1 to 0.67|, thin lines) and strong correlations (“r” scores from ≥|0.67|, thick lines). Common correlations axis is identified by green lines and selective correlation axes observed at each age range are underscored by orange lines. The node sizes are proportional to the number of strong correlations between cell subsets. Cell subsets presenting at least 1 strong correlation are underscored by dark green nodes. The number of total and selective correlations axes between cell subsets at each age range as well as the number of common correlation axes along all age groups are provided in the figure. Circular backgrounds underscore the proportional contribution of each cell cluster to the overall connectivity at distinct age groups.

Integrative network analysis was performed to estimate the neighborhood connectivity profile between memory T-cell subsets and B-cells along childhood and adolescence (Fig. 6). The networks were constructed based on the correlation analysis between values of memory T and B-cell subsets obtained at each age range. The number of age-selective/total correlations were lower at childhood (9^Mths^–1^Yr^ = 6/32; 2^Yrs^ = 4/35; 3–4^Yrs^ = 0/26; 5-7^Yrs^ = 6/37 and 8–10^Yrs^ = 3/34), reaching higher values at 11–18^Yrs^ (14/44) (Fig. 6). The analysis of common correlations along all age groups identified a set of 19 conserved axis, integrating naive cells, memory T and B-cell subsets (Supplementary Fig. 2).

### Integrative networks of memory T and B-cells along healthy childhood and adolescence according to sex

**Figure 7:**
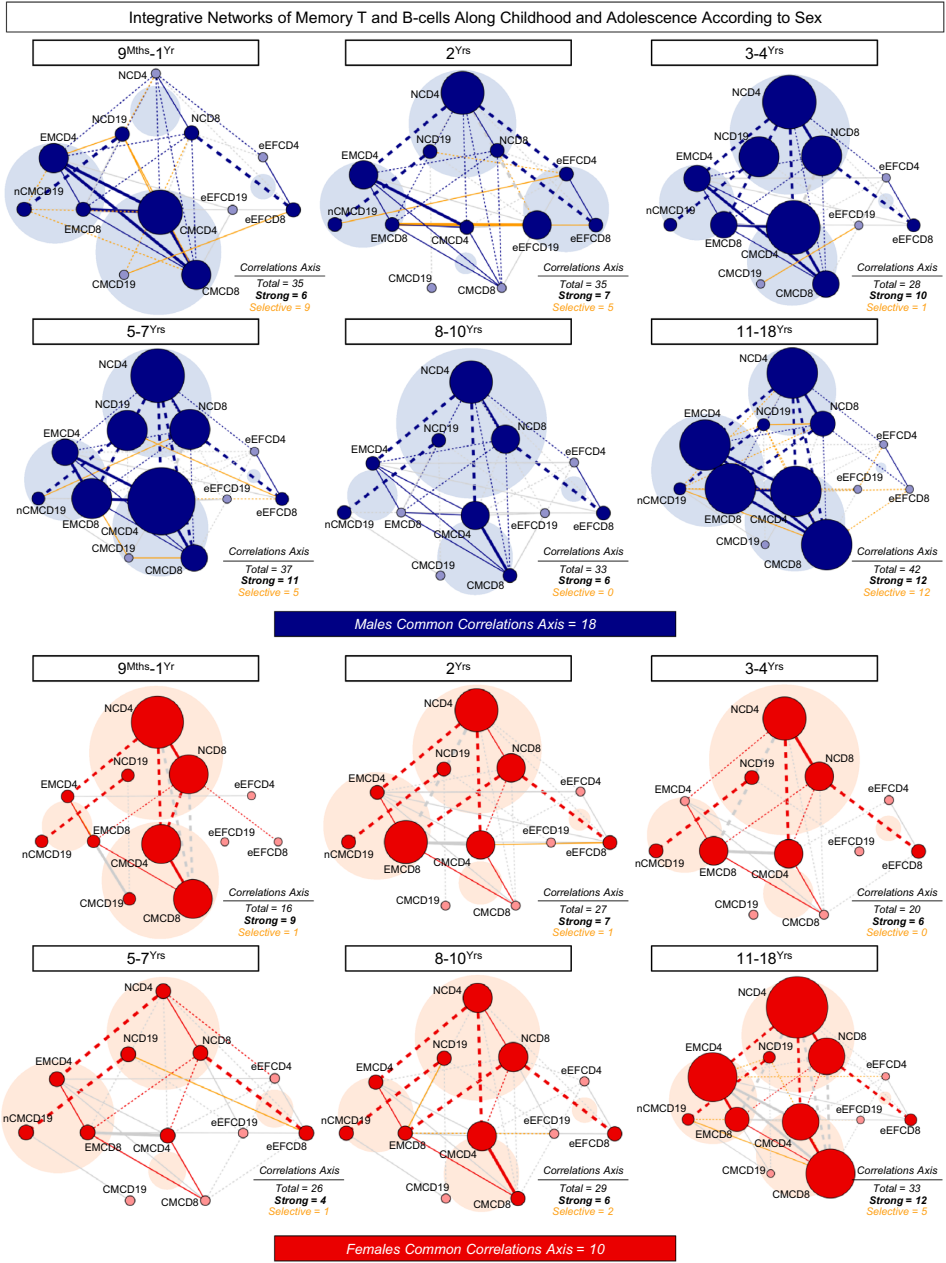
Integrative networks of memory T and B-cells along childhood and adolescence according to sex. Comprehensive networks were built for memory T-cell subsets (CD4^+^ and CD8^+^) and B-cells from healthy children and adolescents, categorized according to sex (Males (Blue color bar), n = 408 and Females (Red color bar), n = 404) followed by age stratification into distinct groups (9^Mths^–1^Yr^, n = 62, n = 73; 2^Yrs^, n = 67, n = 80; 3–4^Yrs^, n = 67, n = 62; 5–7^Yrs^, n = 72, n = 61; 8–10^Yrs^, n = 71, n = 69 and 11–18^Yrs^, n = 69, n = 59, respectively). Data analyses were carried out by Spearman rank tests as described in Material and Methods. Networks were built using cluster layouts comprising four groups of memory cell phenotypes, including: NAIVE (N), Early EFFECTOR (eEF), CENTRAL/NON-CLASSICAL (CM/nCM) and EFFECTOR/CLASSICAL MEMORY (EM/CM) subsets. Connecting edges identify positive (continuous line) and negative (dashed line) correlations. The line thickness illustrates the correlation strength, comprising weak/moderate (“r” scores from |0.1 to 0.67|, thin lines) and strong correlations (“r” scores from ≥|0.67|, thick lines). Common correlations axes are identified by blue/red lines and selective correlation axis observed at each age range are underscored by orange lines. The node sizes are proportional to the number of strong correlations between cell subsets. Memory cell phenotypes participating in at least 1 strong correlation are underscored by dark green nodes. The number of total and selective correlations between cell subsets at each age range as well as the number of common correlation axes along all age groups are provided in the figure. Circular backgrounds underscore the proportional contribution of each cell cluster to the overall connectivity at distinct age groups.

The networks of memory T and B-cell subsets along childhood and adolescence were assembled according to sex and the results are presented in Fig. 7. In general, males presented higher number of correlations than females. However, regardless of sex, the number of age-selective/total correlations were lower at childhood (9^Mths^–1^Yr^ = 9/35; 2^Yrs^ = 5/35; 3–4^Yrs^ = 1/28; 5–7^Yrs^ = 5/37 and 8–10^Yrs^ = 0/33 for males and 9^Mths^–1^Yr^ = 1/16; 2^Yrs^ = 1/27; 3–4^Yrs^ = 0/20; 5–7^Yrs^ = 1/26 and 8–10^Yrs^ = 2/29 for females), reaching higher values at 11–18^Yrs^ (12/42 for males and 5/33 for females) (Fig. 7). The analysis of common correlations along all age groups identified a set of conserved axes integrating naive cells, memory T and B-cells, with higher number of conserved connectivity observed in males as compared to females (18 and 10, respectively) (Supplementary Fig. 2).

## Discussion

There are several pieces of evidence that point out for the development and the ageing processes as non-linear events influencing the immune system, with changes occurring with distinct magnitudes and rhythms in childhood, adolescence, adulthood, and advanced ages. These changes are characterized by waves and are reflected in the production of soluble mediators, such as IL-1β, IL-12, IL-15, IL-7 and IL-2, as well as in the composition of memory T and B cell subsets^5,10–13^.

Ageing begins in the moment we start to live and we can consider that the development process at childhood and adolescence is part of it. In this sense, combining the scientific knowledge on the changes that occur in the immune system during childhood, with those occurring in later ages, can provide insights for the understanding of the ageing process throughout lifetime. During childhood, the immune system gradually matures due to the exposure to infections and vaccinations. As a result of these challenges, the individual develops an expanding repertoire of specific memory immune cells that guarantees long standing protection^2^. Aiming at providing a better comprehension of the changes in the immune system regarding cellularity and of the rhythms in which they occur, the present study investigated the proportion of T and B-cell subsets during childhood and adolescence.

In general, changes in the percentages of T and B-cells were observed during childhood. Memory T-cells expand throughout childhood and adolescence, while the naïve cell compartment contracts. This pattern is similar for CD4^+^ and CD8^+^ T-cells as well as B-cells. In agreement, previous studies have reported similar overall shift of naïve T and B-cells to mature cells as we age^5,13^. This phenomenon could be characterized as a development of a robust immune response after multiple exposure to external challenges. It is important to mention that differences were observed between the rhythms of memory T and B-cells along childhood development. While there was a clear association between the changes in all memory T-cell subsets with age continuous, our findings demonstrated that only NCD19 and CMCD19 B-cells presented an association with age continuum along childhood development. Amongst B-cell subsets, our data demonstrated a peak of naïve cells around 3–4^Yrs^ and 5–8^Yrs^, contrasting with classical memory, which presented higher values at 9^Mths^–1^Yr^ and 11–18^Yrs^. The analysis of predicted probability along age continuum demonstrated an opposite profile between the progressive waning of NCD19 and increasing frequency of CMCD19. Previous reports by Schatorjé et al.^14^ and Duchamp et al.,^15^, carried out in non-endemic areas for parasitic/infectious diseases, demonstrated a progressive decline in naïve B-cells (CD27^-^IgD^+^) and an increase of memory B-cells (CD27^+^IgD^-^) along childhood development. As the age ranges included in our study differ from those employed by Schatorjé et al.^14^ and Duchamp et al.^15^, our results could not be directly compared with their previous reports. However, our findings of predicted probability corroborated the rhythm of B-cell subsets identified by them during childhood development^14,15^. Besides the differences in the age ranges employed in our study, other factors that may account for the discrepancies would be the intrinsic factors associated with the immunobiography of the study populations living in endemic and non-endemic areas for parasitic/infectious diseases. During childhood, the changes in the lymphocyte compartment and the relative percentage of memory B-cells over time reflect the infant exposure to antigenic stimuli during parasitic/infectious diseases. Depending on the pathogen, distinct memory B-cells are developed, which constitutes a target for pathogen-induced subversion. This may lead to altered memory B-cell phenotypes^16^. Another point that should be considered is that infants have significantly higher numbers of peripheral CD19^+^ B cells as compared to adults, and unlike T-cells, the B-cell compartment displays a distinct scattering profile along age ranges. While the frequencies of T-cells show a more homogeneous distribution between minimum and maximum values within a given age range (2.7 times difference), a broader variation (23 times difference) is observed for B-cells among subjects within each age range^17^. Together, these phenomena may explain the apparent divergent rhythms of memory T and B-cells along during childhood development reported amongst studies carried out in endemic and non-endemic areas for parasitic/infectious diseases.

Some of the memory CD4^+^ and CD8^+^ T and B-lymphocyte subsets showed high connectivity in all age groups (overall study population, males, and females). In this sense, NCD4^+^ and CMCD4^+^ seemed to interact more frequently with other cell subsets. These results suggest that these cells play an important role when the immune system is challenged. CMCD4^+^ are restricted to blood and lymphoid organs, however, they provide an adaptive protection through multiple pathways^18^. This protective response is influenced by the challenges occurring in the external milieu such as infections or vaccination^18^. The increased NCD4^+^ phenotype may be associated with the age of the individuals since during childhood and adolescence they undergo a period of complex immunity development. Thus, naive cells are recruited and, thus, undergo differentiation into memory phenotypes to build a robust immune system.

Our study suggests that normal development during childhood does not only depend upon changes in memory T and B-cell subsets, but cell-to-cell interaction. We demonstrated that by calculating their connectivity during development. The neighborhood connectivity and predicted probabilities had specific periods when changes can be identified. Around the age of 10 years, a broad increase of connectivity, in addition to an increased probability of find mature memory cells (eEFCD4^+^, eEFCD8^+^, CMCD4^+^, CMCD8^+^, EFCD4^+^, and EFCD8^+^) were observed, regardless of sex. These alterations could be associated to an endocrine event named gonadarche. The gonadarche consist in the pubertal reactivation of the hypothalamic–pituitary–gonadal axis, which results in development of gonads and posterior sexual development^19^. The average age of onset of this event in females is approximately 10 years of age, while in males this event occurs around 11 years of age^20–22^. In spite of these relevant findings, it is important to mention that changes in the connectivity based on correlation analysis do not necessarily result or reflect on changes in biological connections and pathways and further studies are yet required to better understand the relationship between these findings and changes in cellular functions.

Sexual hormones can influence the immune responses by binding nuclear receptors or membrane-bound receptors that initiate signal transduction pathways in immune cells^8,23,24^. Both innate and adaptive immune cells express hormone receptors as reported in hematopoietic stem cells, natural killer cells, plasmacytoid dendritic cells and monocytes as well as T and B-lymphocytes^23,24^. Thompson and colleagues^25^ have reported that adolescents, both males and females, presented significant changes in the proportion of lymphocytes pre- and post-puberty and, that these changes during puberty may occur in response to sex hormones. Little is known about the mechanisms of immunomodulation by sexual hormones. Androgens can induce thymic involution by reducing the entry of bone marrow-derived stem cells in the thymus and by inducing the loss of thymic epithelial cells^26^. Lymphocytes are also influenced by androgens and, therefore, it is possible that the increase in connectivity observed after the age of 11 is the result of the interaction of memory T-cell subsets (CD4^+^ and CD8^+^) and B-cells with sex hormones. These processes occur as there are changes in the production, secretion and serum concentration of these endocrine components at this early period of lifetime. Along with sexual hormonal production, anthropometric data and previous infectious/parasitic diseases are aspects that could impact the immune response shaping the immune system over the years. However, in the present investigation, we do not have measure these variables. Thus, studies that better characterize the dynamics of changes in the profile of memory T and B-cells and other biological factors and their interactions can further contribute to a clear understanding of the early years of immunobiography.

This is a pioneer investigation assessing the development of memory T and B-cell subsets during healthy childhood and adolescence in Minas Gerais, Brazil. However, the present study has some limitations. Despite the pioneering approach of this exploratory investigation, the observational design with multiple comparisons along ages during childhood development without corrections for the presence of parasitic/infectious diseases that may influence the profile of memory T and B-cells; nutritional aspects and hormonal measurements or other confounding variables constitute a limitation that may interfere in the interpretations of our findings. In the present study, we did not collect the hematological records to obtain the total lymphocyte counts for the study population and, therefore, the data were reported exclusively as relative percentages of cells within gated lymphocyte subsets and not as absolute cell numbers. Another limitation is that this is a single-center study in Belo Horizonte-Minas Gerais state, Brazil and the generalization of our findings to other epidemiological scenarios should be considered with caution.

Altogether, our findings demonstrated that the cell rhythmic signatures described here revealed a complex interaction between these cell subsets. Furthermore, the data highlighted the important changes that occurred during childhood development with rhythmic changes appearing at around 2^Yrs^, with predominance of naive cells. Additionally, the second and more prominent wave occurred around the age 8-10^Yrs^, with the prevalence of memory phenotypes. The neighborhood connectivity profile analysis also indicated a peak of integrative network connections at 11–18^Yrs^, possibly reflecting the onset of maturation of the reproductive system, hormonal shifts, and their influence in the immune system. All in all, our results provided new insights into immunobiography and a comprehensive understanding of interactions amongst the cell subsets during the onset of the ageing process that encompasses childhood and adolescence.

## Population, material and methods

### Study population

This is a descriptive-observational investigation carried out in Belo Horizonte—Minas Gerais, Brazil, comprising 812 healthy children and adolescents (408 males and 404 females), age ranging from 9 months to 18 years. The participants were enrolled during prior routine vaccination at primary care medical centers. Heparinized whole peripheral blood samples (5–7 mL) were collected at outpatient health basic units after pre-screening carried out by a trained nurse professional to exclude subjects with vaccination records within 30 days prior blood collection, pregnant women, HIV carriers, patients with chronic immune-mediated diseases and patients under immunosuppressive therapy, with hemoglobinopathies, history of blood transfusion, or treatment with hyperimmune serum up to 90 days prior to blood collection.

The study population (ALL, n = 812) was clustered into six consecutive age groups, referred as: 9^Mths^–1^Yr^ (n = 135); 2^Yrs^ (n = 147); 3–4^Yrs^ (n = 129); 5–7^Yrs^ (n = 133); 8–10^Yrs^ (n = 140); 11–18^Yrs^ (n = 128). Subsequently, subjects were sub-grouped according to sex (Males, n = 408 and Females, n = 404) followed by age stratification into sequential age groups (9^Mths^–1^Yr^, n = 62; n = 73; 2^Yrs^, n = 67; n = 80; 3–4^Yrs^, n = 67; n = 62; 5–7^Yrs^, n = 72; n = 61; 8–10^Yrs^, n = 71; n = 69 and 11–18^Yrs^, n = 69; n = 59 for male and female, respectively). The Supplementary Table 1 summarizes the number of subjects as well as the age distribution (median; min and max) for each study group.

### Ethical statements

This study protocol was submitted and approved by the Ethics Committee for research with human beings at Instituto René Rachou–Fiocruz-Minas (C.A.A.E 0023.0.245.000-10). The approved protocol fulfills the Helsinki declaration and was in accordance with the Code of Ethics of the World Medical Association for human studies. Written informed consent was obtained from parents and/or legal guardians of the participants before inclusion in the present investigation.

### Flow cytometric immunophenotypic staining of memory T and B-cells

The immunophenotypic analysis of memory T and B-cell subsets was carried out as previously described by Campi-Azevedo et al.^27^. Briefly, peripheral blood mononuclear cells (PBMC) were obtained from heparinized whole blood samples upon Ficoll-Hypaque density gradient centrifugation. Aliquots of 5.0 × 10^5^ cells/well were incubated with RPMI-1640 medium supplemented with 5% heat-inactivated normal human AB serum, 2 mM l-Glutamine and 3% antibiotic/antimycotic at 37 °C in a 5% CO_2_ for 144 h. Following, cells were stained with a mix of monoclonal antibodies (mAbs) to identify memory T-cell subsets (anti-CD3/2P34-2/APC-Cy7, anti-CD4/RPA-T4/FITC, anti-CD8/SK1/PerCP-Cy5.5, anti-CD27/M-T271/PE, anti-CD45RO/UCHL1/PE-Cy7) and memory B-cells (anti-CD19/HIB19/PerCP, anti-IgD/IA6-2/FITC and anti-CD27/M-T271/PE) (BD Biosciences, San Jose, CA, USA). Stained cells were washed once and fixed with FACS fix solution (10 g/L paraformaldehyde, 10.2 g/L sodium cacodylate, 6.65 g/L sodium chloride (Sigma, St. Louis, MO, USA)). At least 100,000 lymphocytes/sample were acquired on a LSR Fortessa Flow Cytometer (BD Biosciences, San Diego, CA, USA).

Distinct gating strategies were employed to analyze memory T and B-cell subpopulations, as illustrated in Supplementary Fig. 1. The gating strategies were set up as previously described^28–30^. The FlowJo software (version 10.7.2, BD Biosciences, San Diego, CA, USA) was employed for the phenotypic analysis used to quantify T and B-cell subsets as follows: memory CD4^+^ and CD8^+^ T-cell subsets comprised NAÏVE/N (CD45RO^−^CD27^+^), Early EFFECTOR/eEF (CD45RO^−^CD27^−^), CENTRAL MEMORY/CM (CD45RO^+^CD27^+^) and EFFECTOR MEMORY/EM (CD45RO^+^CD27^−^) phenotypes. B-cells (CD19^+^) subsets comprised NAÏVE/N (CD27^−^IgD^+^), Early EFFECTOR/eEF (CD27^−^IgD^−^), NON-CLASSICAL MEMORY/nCM (CD27^+^IgD^+^) and CLASSICAL MEMORY/CM (CD27^+^IgD^−^) phenotypes. The final results of lymphocyte memory subsets are presented as relative frequency of gated cells (% within gated CD4^+^, CD8^+^ or CD19^+^ gated cells).

### Statistical analysis

Prism 9.0.1 software (GraphPad Software, San Diego, USA) was used for statistical analysis. Data normality test was assessed by the Shapiro–Wilk test. Comparative analysis between pairs of adjacent age groups was carried out by Mann–Whitney test, considering a threshold of p < 0.05 for statistical significance.

The analysis of association between memory T and B-cells with childhood and adolescence was carried out upon data categorization using cut-offs established at the 75^th^ percentile of each data set empirical distribution. Logistic regression model was employed to quantify the strength of association between the frequency of memory CD4^+^ or CD8^+^ T and B-cells and age, as well as putative variations by sex. Based on the adjusted model, the predicted probabilities of subjects to present a given phenotype of memory T or B-cells subsets in high frequency were estimated according to age continuum (months). The logistic regression model fit was assessed by the Hosmer–Lemeshow test. The analysis was performed for the general sample (ALL) and stratified by sex (Males and Females), considering a level of 5% of significance. All analysis was performed in Stata software, version 14.0.

Overall signatures of memory T and B-cells subsets were built by first converting the original data expressed as continuous variables (percentage of gated cells) into categorical data (proportion) using the global median values establish for each memory T and B-cells to estimate the proportion of subjects above the cut-off edges. The memory T and B-cells with proportion of subjects above the 50th percentile was underscored on radar charts assembled using the Microsoft Excel (Version 0.11.2) and orbital plots built using the Python software (Version 3.9.0.). Comprehensive heatmap constructs were assembled using the equalized distribution of subjects with results above the global median cut-offs employing the heatmap function of the Seaborn data visualization library (Version 0.11.2) of Python software (Version 3.9.0.).

Correlation analysis was carried out using the Spearman rank test and the “r” scores of significant correlations (p < 0.05) employed to generate integrative networks using the systems biology approaches with the Cytoscape open-source platform (available at https://cytoscape.org). The category cluster layout was chosen for arranging the networks of memory T and B-cell subsets. The memory cell phenotypes participating of at least one strong correlation (“r” scores from ≥|0.67|) were highlighted by darker nodes and the connecting edges underscored by thick dark lines. The number of connections between cell subsets was calculated and used for comparative analysis between age ranges.

### Supplementary Information

Supplementary Information.

## Data Availability

The results included in the present study are available from the corresponding authors [OAMF and KCLT] upon request.
