# Supplementary material for: Rhythmic profile of memory T and B-cells along childhood and adolescence
Source: Sci Rep. 2023 Nov 28;13:20978. doi: 10.1038/s41598-023-48115-3 (PMC10684863; doi:10.1038/s41598-023-48115-3)
Supplement: Supplementary file 1 — Supplementary Information. [file 41598_2023_48115_MOESM1_ESM.pdf]

Rhythmic profile of memory T and B-cells along childhood and adolescence

Joaquim Pedro Brito-de-Sousa[1§]; Maria Luiza Lima-Silva[2§], Ismael Artur da Costa-Rocha[2§], Luiz Roberto Alves de Oliveira Júnior[2], Ana Carolina Campi-Azevedo[2], Vanessa Peruhype-Magalhães[2], Josiane da Silva Quetz[2,3], Jordana Grazziela Alves Coelho-dos-Reis[4], Christiane Costa-Pereira[2], Cristiana Couto Garcia[2], Lis Ribeiro do Vale Antonelli[2], Cristina Toscano Fonseca[2], Jandira Aparecida Campos Lemos[5], Juliana Vaz de Melo Mambrini[2], Elaine Maria Souza-Fagundes[4], Andréa Teixeira-Carvalho[2], Ana Maria de Caetano Faria[6], Angelica Oliveira Gomes[7], Karen Cecília de Lima Torres[2,3#*], Olindo Assis Martins-Filho[1,2#*].

Representative Flow Cytometry Gating Strategies for Phenotypic Analysis of Memory T and B-cells

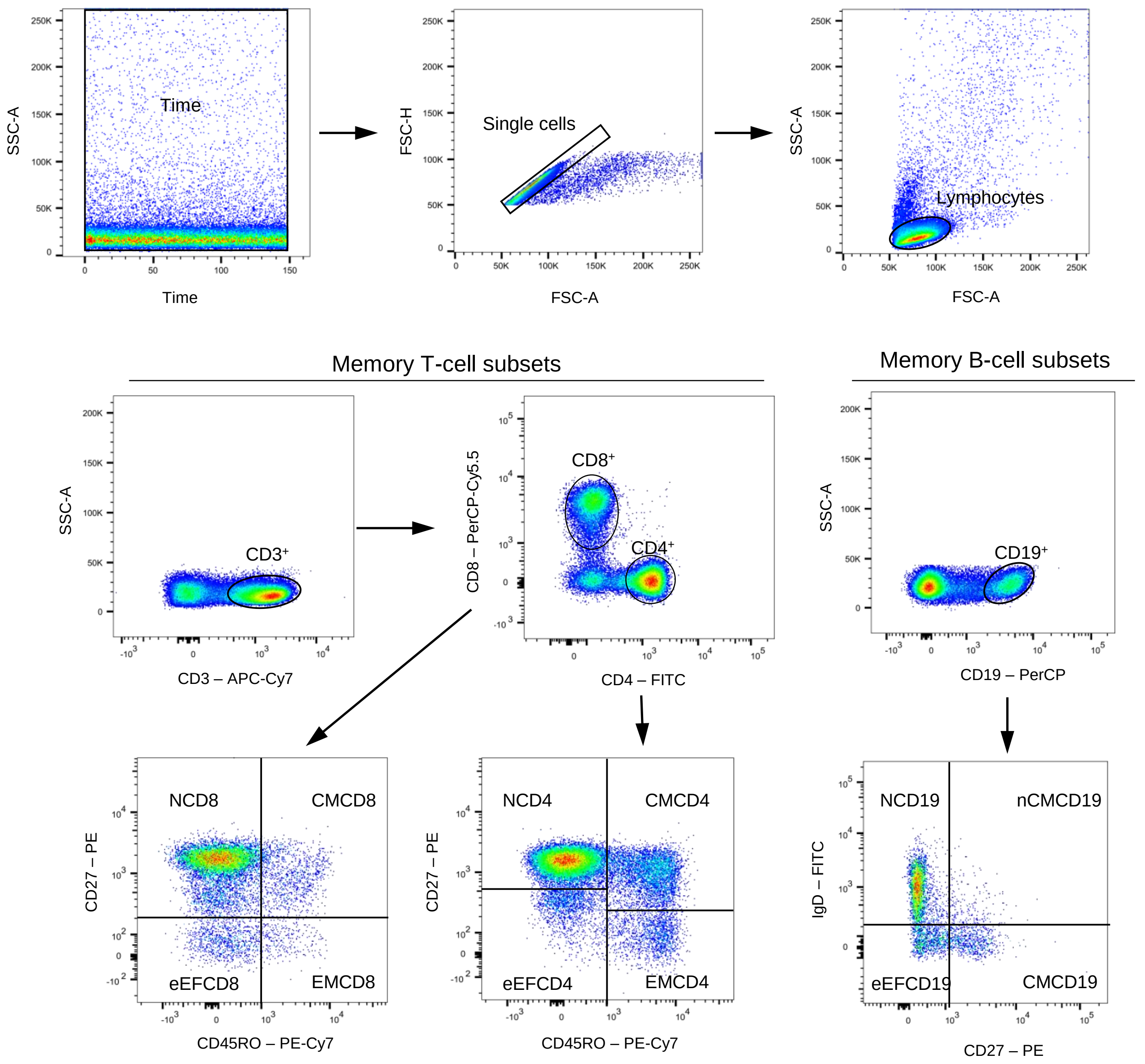

Supplementary Figure 1 – *Representative flow cytometry gating strategies for phenotypic analysis of memory T and B-cells.* Immunophenotypic staining of PBMC were carried out by flow cytometry as described in Material and Methods. Data acquisition was carried out in a LSR Fortessa Flow Cytometer running a total of 100,000 lymphocytes per sample. The FlowJo™ Software (version 10.7.2) was employed for the analysis of flow cytometry data. Representative pseudocolor density plots illustrate the gating strategies used to analyze memory T and B-cell subsets. Gating strategies start with events selection by Time *vs* Side Scatter Area (SSC-A) to assess the acquisition quality of continuous laser scatter profile. Following, single cells were gated based on Forward Scatter Area (FSC-A) *vs* Forward Scatter Height (FSC-H) parameters. Thereafter, lymphocytes were selected based on their size *vs* granularity morphometric properties (FSC-A *vs* SSC-A). Phenotypic analysis of memory T and B-cell subsets were further performed using bidimensional fluorescence pseudocolor density plots. The quantification of memory $CD4^+$ and $CD8^+$ T-cell subsets were carried out according to the CD45RO and CD27 immunostaining to identify: naïve – N ($CD45RO^-CD27^+$), early effector – eEF ($CD45RO^-CD27^-$), central memory – CM ($CD45RO^+CD27^+$) and effector memory – EM ($CD45RO^+CD27^-$). The analysis of memory B-cell subsets were performed based on the immunostaining profile of IgD and CD27 to identify: naïve – N ($CD27^-IgD^+$), early effector – eEF ($CD27^-IgD^-$), non-classical memory – nCM ($CD27^+IgD^+$) and classical memory – CM ($CD27^+IgD^-$).

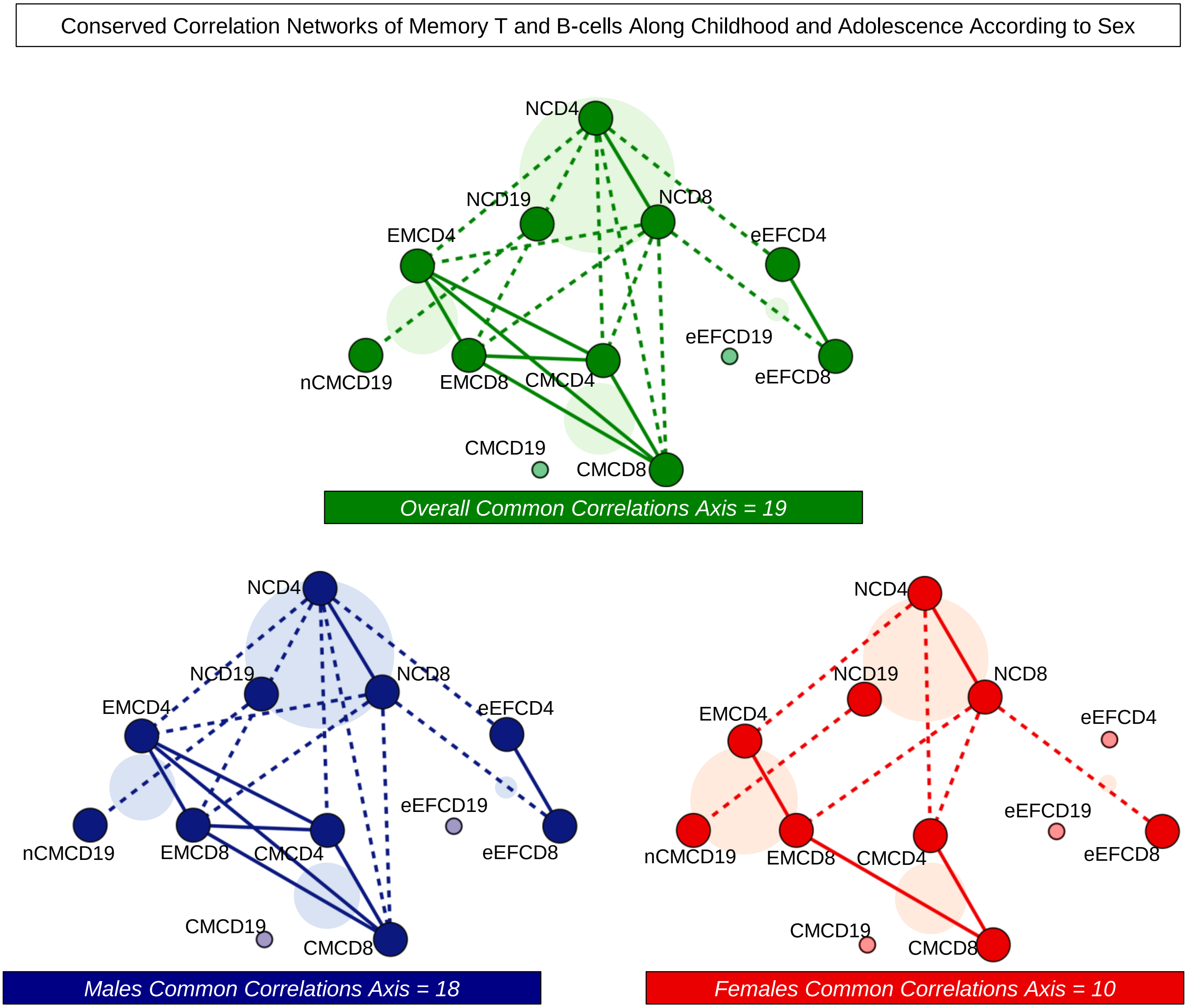

Supplementary Figure 2

Supplementary Figure 2 – *Conserved Correlation Networks of Memory T and B-cells Along Childhood and Adolescence According to Sex*. Networks were built for memory T-cell subsets ($CD4^+$ and $CD8^+$) and B-cells from healthy children and adolescents (ALL (Green), n=812), categorized according to sex (Males (Blue), n=408 and Females (Red), n=404). Data analyses were carried out by Spearman rank tests and triangle correlation matrices were built to compile the "r" scores (scaled from −1 to +1) of significant correlations ($p<0.05$) between memory T-cell subsets and B-cells employing a blue and red gradient color key. Networks were built using cluster layouts comprising four groups of memory cell phenotypes, including: NAIVE (N), Early EFFECTOR (eEF), CENTRAL/NON-CLASSICAL (CM/nCM) and EFFECTOR/CLASSICAL MEMORY (EM/CM) subsets. Connecting edges identify positive (continuous line) and negative (dashed line) correlations. Common correlations axes are represented by color lines. The node sizes are proportional to the number of strong correlations between cell subsets. Memory cell phenotypes participating in at least 1 strong correlation are underscored by dark color nodes. The number of common correlations axes between cell subsets along all age groups are provided in the figure. Circular backgrounds underscore the proportional contribution of each cell cluster to the overall connectivity.

Supplementary Table 1: Study population

Age Groups	ALL		Males		Females	
	n	Median Age (Min-Max)	n	Median Age (Min-Max)	n	Median Age (Min-Max)
9^{Mths}-1^{Yr}	135<br>(100%)	0.9<br>(9^{Mths}–1.5^{Yrs})	62<br>(46%)	0.9<br>(9^{Mths}–1.5^{Yrs})	73<br>(54%)	0.9<br>(9^{Mths}–1.5^{Yrs})
2^{Yrs}	147<br>(100%)	1.9<br>(1.6^{Yrs} –2.5^{Yrs})	67<br>(46%)	2.0<br>(1.7^{Yrs} –2.5^{Yrs})	80<br>(54%)	1.9<br>(1.6^{Yrs} –2.5^{Yrs})
3-4^{Yrs}	129<br>(100%)	3.0<br>(2.6^{Yrs} –4.3^{Yrs})	67<br>(52%)	3.0<br>(2.6^{Yrs} –4.3^{Yrs})	62<br>(48%)	2.9<br>(2.6^{Yrs} –4.2^{Yrs})
5-7^{Yrs}	133<br>(100%)	5.1<br>(4.7^{Yrs} –7.5^{Yrs})	72<br>(54%)	5.1<br>(4.7^{Yrs} –7.5^{Yrs})	61<br>(46%)	5.0<br>(4.8^{Yrs} –6.8^{Yrs})
8-10^{Yrs}	140<br>(100%)	8.0<br>(7.7^{Yrs} –10.5^{Yrs})	71<br>(51%)	8.0<br>(7.7^{Yrs} –10.5^{Yrs})	69<br>(49%)	8.0<br>(7.8^{Yrs} –10.5^{Yrs})
11-18^{Yrs}	128<br>(100%)	11.1<br>(10.6^{Yrs} –17.5^{Yrs})	69<br>(54%)	11.1<br>(10.6^{Yrs} –17.5^{Yrs})	59<br>(46%)	11.1<br>(10.6^{Yrs} –15.1^{Yrs})
